# Entropic Confinement in String-Net Models: An Analogue Study via SU(2)_k_ Fusion Categories

**DOI:** 10.3390/e28091017

**Published:** 2026-09-11

**Authors:** Xiaodong Yang

**Affiliations:** 1 CGNPC Uranium Resources Co., Ltd., Beijing 100072, China; yangxiaodong160@163.com; 2 School of Physics Science and Technology, Lanzhou University, Lanzhou 730000, China

**Keywords:** QCD, color flux tube, entanglement entropy

## Abstract

Recent lattice studies have revealed that the color flux tube between static quark–antiquark pairs exhibits an excess entanglement entropy (flux-tube entanglement entropy, FTE^2^) that scales linearly with the quark separation L. In this paper, we demonstrate similar behavior in a string-net model based on $SU(2{)}_{k}$ fusion categories, where the nontrivial object j=1/2 (analogous to color charge) cannot exist in isolation due to the fusion rules, naturally exhibiting “confinement”. We compute the entanglement entropy of the flux tube connecting two j=1/2 objects using the microcanonical (equal-weight) prescription S(R)=ln(dim(Hom(R))) and find an entropy density $\sigma_{k}=\ln d_{1/2}=\ln(2\cos\frac{\pi }{k+2})$. For k=3, the category reduces to the Fibonacci case, yielding an entropy density σ_3_ = ln φ ≈ 0.4812 (φ is the golden ratio), which is qualitatively comparable in magnitude to the scale inferred from lattice studies and the entropy surface mechanism. Under a thermalization assumption for the fusion-channel degrees of freedom, minimizing the free energy $F=(J-T\sigma_{k})L$ yields a confinement–deconfinement transition at $\left(T_{c}=J/\sigma_{k}\right)$, which is first-order-like (tension sign reversal) rather than a continuous critical transition. The parameter k offers a tunable knob, making the $SU(2{)}_{k}$ family a computable laboratory for entropic confinement. The predicted entropy-density jump can be directly tested in quantum simulator platforms (e.g., Rydberg arrays or superconducting circuits) that realize Fibonacci anyonic models.

## 1. Introduction

The nature of color confinement in Quantum Chromodynamics (QCD) remains one of the deepest puzzles in theoretical physics [1,2]. Recently, the quantum information perspective has achieved important progress. Mamo [3] proposed an entropy surface mechanism, arguing that confinement originates from the sign reversal of the entanglement entropy gradient at a critical radius. Amorosso et al. [4,5] computed the flux-tube entanglement entropy (FTE^2^) in (2+1)-dimensional SU(2) lattice gauge theory and found that the internal color entanglement entropy grows linearly with the quark separation L, providing direct evidence for the entropic nature of confinement. Takahashi and Kanada–En’yo [6] also studied entanglement entropies of static quark–antiquark correlations in lattice QCD.

However, these numerical results have not yet been translated into an intuitive conceptual model. We still lack a simple and understandable physical picture of why quarks are confined. These developments raise a fundamental question: can the linear growth of flux-tube entanglement entropy be reproduced in a simple model?

This paper attempts to answer this question within a string-net model based on $SU(2{)}_{k}$ fusion categories. The $SU(2{)}_{k}$ fusion category contains k+1 simple objects labeled by spin j=0,1/2,1,3/2,…,k/2, with fusion rules that naturally imply that the j=1/2 object cannot exist in isolation—precisely a categorical analog of “confinement”. For k=3, the category reduces to the Fibonacci category, the simplest non-Abelian fusion category.

It is important to clarify the scope of this work. The $SU(2{)}_{k}$ string-net model is not a first-principles reduction of QCD; rather, it is a minimal analogue model that captures the algebraic essence of non-Abelian fusion and its entropic consequences. Our comparison with lattice QCD results is therefore at the level of qualitative features (linear growth of entanglement entropy and a temperature-driven transition), not quantitative matching. This analogue perspective enables exact solvability and provides a bridge between topological order and confinement physics.

In string-net models, the topological entanglement entropy of a region R is defined as the logarithm of the dimension of the Hilbert space of anyon excitations on the boundary [7,8]:

S(R)=ln(dim(Hom(R)))

where Hom(R) is the vector space of legal fusion channels from the interior of R to the exterior. This definition directly reflects the non-local entanglement structure of topological order. For a flux tube connecting two j=1/2 anyons, the internal degrees of freedom are described by a fusion tree along the tube, and the total number of states corresponds to the number of fusion channels fusing L copies of j=1/2 to the identity. Thus, the flux-tube entanglement entropy is $S_{flux}(L)=\ln N(L)$, where N(L) is the number of fusion channels.

Before proceeding, a few clarifications are in order regarding the lattice results [4,5]. First, those calculations were performed in (2+1)-dimensional SU(2) gauge theory without dynamical fermions, not in SU(3) QCD. Second, the entanglement entropy studied in [4,5] is defined for a spatial region that partially overlaps the flux tube (a slab geometry), rather than the one-dimensional anyon chain considered here. Our comparison is therefore at the level of qualitative behavior—the linear growth of entanglement entropy with separation—rather than a direct reproduction of the specific ${FTE}^{2}$ quantity. Third, lattice simulations [4,5] indicate a linear growth of the entanglement entropy with separation, with a slope that in natural units is on the order of a few tenths. The entropy surface mechanism of Mamo [3] also points to an entropy density of comparable magnitude. These estimates provide a reasonable reference scale for the flux-tube entropy density, against which our model results can be compared qualitatively.

Recently, Du et al. [9] have experimentally confirmed that chiral graviton modes in fractional quantum Hall systems originate from the geometric oscillations of emergent partons, providing direct evidence that geometry can emerge from quantum many-body correlations.

The present work generalizes our previous analysis restricted to the Fibonacci case [10] to the entire $SU(2{)}_{k}$ family, providing a tunable parameter k that allows a systematic study of the k-dependence of the entropic force and the deconfinement temperature. It should be emphasized that the comparison with lattice QCD is qualitative, intended to illustrate the conceptual mechanism rather than to provide a precise numerical match. Moreover, the finite-temperature phase transition derived below is obtained under a thermalization assumption for the fusion-channel degrees of freedom, which we explicitly state and discuss in Section 4.

The structure of this paper is as follows. Section 2 introduces the basic structure of $SU(2{)}_{k}$ fusion categories and shows how confinement naturally emerges from the fusion rules. Section 3 defines the entanglement entropy using the topological definition, computes the entropy density of the flux tube, and discusses the asymptotic behavior via transfer matrix methods. Section 4 derives the confinement–deconfinement phase transition through free energy minimization and explicitly states the thermalization assumption. Section 5 presents a heuristic analogy between $SU(2{)}_{k}$ fusion categories and the representation category of SU(3). Section 6 discusses the physical implications, including the parameter k as a control knob, the significance of the golden ratio, support for the entropy surface mechanism, a comparison with related categorical models (Ising, Fibonacci, and other string-net approaches), testable predictions, open questions about the reference state of entropy, and limitations of the model. Section 7 concludes the paper with a summary of findings and an outlook for future work. A numerical verification scheme for the string-net model is provided in Appendix A.

## 2. $SU(2{)}_{k}$ Fusion Categories and the Flux-Tube Setup

### 2.1. Simple Objects and Fusion Rules

The $SU(2{)}_{k}$ fusion category contains k+1 simple objects labeled by spin j=0,1/2,1,3/2,…,k/2, as discussed in the context of SU(2) coset constructions [11] and quantum group fusion rules [12]. The object j=0 is the unit object **1** (vacuum). We focus on k≥2, since k=1 corresponds to an Abelian category ($d_{1}{/}_{2}=1$) where the flux-tube entanglement entropy does not grow linearly with length, inconsistent with the non-Abelian nature of QCD color flux tubes.

The fusion rule is

(1) $$j_{1}⊗j_{2}={⨁}_{j=|j_{1}-j_{2}|}^{\min(j_{1}+j_{2},k-j_{1}-j_{2})}j\;$$

where the truncation condition reflects the non-Abelian structure of the category.

### 2.2. Quantum Dimensions and Total Quantum Dimension

The quantum dimension of object j is

(2) $$d_{j}=\frac{\sin\left(\frac{(2j+1)\pi }{k+2}\right)}{\sin\left(\frac{\pi }{k+2}\right)}$$

In particular,

(3) $$d_{0}=1,d_{1/2}=2\cos\left(\frac{\pi }{k+2}\right)$$

The total quantum dimension of the full SU(2)_k_ category is

(4) $$D_{k}=\sqrt{\sum_{j=0}^{k/2}d_{j}^{2}}=\frac{\sqrt{k+2}}{\sqrt{2}\sin\left(\frac{\pi }{k+2}\right)}$$

This expression holds for k≥2; the k=1 case is Abelian and is excluded.

### 2.3. Categorical Analogue of Confinement 

The fusion rule for two j = 1/2 objects is

(5) $$\frac{1}{2}⊗\frac{1}{2}=0⊕1$$

This implies the following:

(1) A single j=1/2 object **cannot** fuse to the unit object **1** through any fusion process; it cannot appear as an isolated gauge-invariant state.
(2) Two j=1/2 objects **can fuse to 1**, analogously to a $q\bar{q}$ meson singlet.
(3) In a flux-tube geometry with two separated j=1/2 objects at the ends, the total topological charge of the entire system is required to be **1** (a singlet). The internal chain connecting them must therefore contain an even number of j=1/2 objects so that the overall fusion product yields **1**. This even-length chain mimics the color-singlet flux tube between a $q\bar{q}$ pair. A chain with an odd number of 1/2 objects would carry a total charge of 1/2 (or a higher half-integer) and is therefore not a gauge-invariant flux tube.

**Key point**: A single j=1/2 object cannot exist in isolation; it must pair (or form an even-length chain) to yield a total charge of **1**. This is precisely the categorical analog of the statement that colored objects are confined within singlets. We note that $SU(2{)}_{k}$ carries a $Z_{2}$ grading: an odd number of half-integer spins can never fuse to an integer (vacuum). Our flux-tube construction only uses even-length chains, so it respects this grading.

### 2.4. The k=3 Special Case: Fibonacci Category [10]

When k=3, the simple objects are j=0,1/2,1,3/2. Their quantum dimensions are

(6) $$d_{0}=1,\;d_{1/2}=\phi ,\;d_{1}=\phi ,\;d_{3/2}=1$$

where $\phi =(1+\sqrt{5})/2$ is the golden ratio.

The Fibonacci category is the **integer-spin subcategory** generated by j=0 and j=1. In this subcategory, the nontrivial object τ corresponds to j=1, and its fusion rule is

τ⊗τ=1⊕τ

which is the well-known Fibonacci fusion rule [13,14].

Although the fundamental constituent in our flux-tube chain is the half-integer object j=1/2 and the chain must have even length to yield a singlet), the **asymptotic entropy density** of such an even-length chain is determined by the largest quantum dimension appearing in the fusion graph, which is $d_{1/2}=\phi$. Therefore, for k=3, the flux-tube entanglement entropy density coincides numerically with that of the Fibonacci category:

$$\sigma_{3}=\ln\phi \approx 0.4812$$

This coincidence is why our previous work [10] focused on the Fibonacci case; the present generalization to arbitrary k shows that this entropic behavior is actually a special case of the broader $SU(2{)}_{k}$ family.

### 2.5. Flux Tube in the String-Net Model

In the Levin–Wen string-net model [7,8,15], anyon excitations of the $SU(2{)}_{k}$ category correspond to defects in the string-net. Two j=1/2 objects (located at two points in space) are connected by a flux tube whose length L (in units of lattice spacing) corresponds to the quark separation. The internal configuration of the flux tube is described by string-net fluctuations. A comprehensive field-theoretic description can be found in [16].

In the following, we discretize the flux tube as a one-dimensional chain of L sites, each hosting a j=1/2 object, with the total charge fixed to **1** (singlet). This requires L to be even, but the asymptotic entropy density is independent of this parity constraint for L ≫ 1. For a systematic discussion of enriched string-net models and their excitation spectra, see Ref. [17].

### 2.6. Counting Fusion Channels: General Transfer Matrix for Arbitrary k (Revised)

For the $SU(2{)}_{k}$ fusion category, consider a chain of L copies of the j=1/2 object, with the total charge fixed to **1** (the vacuum). Let N(L) be the number of such fusion channels. To count N(L) for general k, we introduce the set of all possible intermediate spins that can appear when fusing a sequence of 1/2 objects. These spins are precisely the simple objects of the category:

{0,1/2,1,3/2,…,k/2}

Define a transfer matrix T of dimension (k+1)×(k+1), whose rows and columns are labeled by these spins. The matrix element $T_{a,b}=1$ if the spin b appears in the fusion product a⊗1/2, and 0 otherwise. In other words, T is the adjacency matrix of the $A_{k+1}$ Dynkin diagram (with nodes labeled by the spins, ordered from 0 to k/2. Explicitly, for two consecutive nodes a and b:

$$T_{a,b}=\left\{\begin{array}{ll} 1, & if\;|a-b|=1/2,\\ 0, & otherwise \end{array}\right.$$

with the truncation that the maximal spin is k/2.

The number of ways to fuse L copies of 1/2 to the singlet (final spin (0)) is then

$$N(L)=(T^{L}{)}_{0,0}\;(\mathrm{for}\;\mathrm{even}\;L)$$

For odd L, the entry $(T^{L}{)}_{0,0}$ vanishes identically due to the $Z_{2}$ grading discussed in Section 2.3. Since our flux tube requires even-length chains to yield a singlet, this restriction does not affect the result.

The matrix T is symmetric and its eigenvalues are known from the representation theory of $SU(2{)}_{k}$ [11,12]:

$$\lambda_{m}=2\cos\left(\frac{m\pi }{k+2}\right),\;m=1,2,\ldots ,k+1$$

The **largest** eigenvalue is

(7) $$\lambda_{\max}=2\cos\left(\frac{\pi }{k+2}\right)=d_{1/2}$$

Therefore, for L≫1

(8) $$N(L)∼\lambda_{\max}^{L}=d_{1/2}^{L}$$

For k=3, the full transfer matrix is 4×4. However, if we restrict to even-length chains starting and ending at spin 0, the relevant subspace reduces to a 2×2 matrix whose eigenvalues are ϕ and −1/ϕ, recovering the Fibonacci recurrence used in our previous work [10]. For general k≥4, the full (k+1)×(k+1) matrix is required, but the final asymptotic result—the entropy density $\sigma_{k}=\ln d_{1/2}$—remains unchanged.

For a general introduction to fusion categories, see [18].

## 3. Entanglement Entropy

### 3.1. Topological Definition of Entanglement Entropy

For the flux-tube excited state in the string-net model, we adopt a microcanonical (equal-weight) prescription for the entanglement entropy of the internal fusion channels. Given a flux-tube segment with a fixed total topological charge, the accessible Hilbert space is spanned by all legal fusion channels. Assuming these channels are equally probable, the entanglement entropy is

(9) $$S(R)=\ln\left(\dim(Hom(R))\right)$$

where Hom(R) is the vector space of legal fusion channels from the interior of the flux-tube segment to its exterior. This is the maximum-entropy value for the given Hilbert-space dimension. We emphasize that this is not the ground-state topological entanglement entropy defined in Refs. [7,8] (which is a constant −lnD), but rather an application of the same “log-dimension” counting principle to the excited flux-tube sector.

### 3.2. Entanglement Entropy of the Flux Tube

Consider a flux tube connecting two j=1/2 anyons, discretized into L lattice sites (physical length L⋅a). In the string-net picture, the flux tube is a chain of L linked anyons with fixed total topological charge **1** (singlet). The number of valid fusion channels N(L) is the number of ways to fuse L copies of j=1/2 to the identity.

For the $SU(2{)}_{k}$ fusion category, the fusion rule $\frac{1}{2}⊗\frac{1}{2}=0⊕1$ implies that the number of channels satisfies a linear recurrence. The asymptotic behavior for L≫1 is dominated by the largest quantum dimension:

(10) $$N(L)∼d_{1/2}^{L},\;d_{1/2}=2\cos\frac{\pi }{k+2}$$

This asymptotic behavior is derived in Section 2.6 using the transfer matrix method.

### 3.3. Asymptotic Behavior and Entropy Density

According to the topological entanglement entropy definition, the entanglement entropy of the flux tube is

(11) $$S_{flux}(L)=\ln N(L)\approx L\ln d_{1/2}+constant$$

Thus, the entropy density is

(12) $$\sigma_{k}\equiv \ln d_{1/2}=\ln\left(2\cos\frac{\pi }{k+2}\right),\;k\geq 2$$

For k=3, Equation (12) gives

(13) $$\sigma_{3}=\ln\phi \approx 0.4812$$

### 3.4. Numerical Comparison with QCD

From the lattice calculation of Ref. [4], the flux-tube entanglement entropy in (2+1)-dimensional SU(2) gauge theory grows linearly with quark separation with a slope estimated to be of order 0.3–0.5 in natural units (the exact value depends on the lattice spacing and the specific entanglement region geometry; see Ref. [4] for details). The entropy surface mechanism discussed in Ref. [3] suggests a similar order of magnitude. Within this approximate range, our model yields $\sigma_{2}=\ln\sqrt{2}\approx 0.3466$ and $\sigma_{3}=\ln\phi \approx 0.4812$, both of which fall within the same order of magnitude.

We stress that this comparison is **qualitative**: the lattice result is for SU(2) gauge theory without dynamical fermions, computed in a slab geometry, whereas our string-net model is a one-dimensional anyonic chain. The agreement at the order-of-magnitude level is therefore meant only to illustrate the conceptual viability of the entropic confinement mechanism, not to constitute a precision test.

For k≠3, the model retains its value as an independent theoretical laboratory, allowing systematic study of how the entropic force depends on the categorical parameter k. The dependence of $\sigma_{k}$ on k is shown in Table 1.

Lattice studies of Rényi entanglement entropy in SU(N) Yang–Mills theories have also been performed for N = 2, 3, 4 [19]. Hamiltonian lattice studies of string dynamics in SU(2) gauge theory have been carried out in [20].

## 4. Free Energy Minimization and the Deconfinement Phase Transition

### 4.1. Finite-Temperature Free Energy: A Thermalization Assumption

In the string-net model, the internal energy of a flux tube of length L (in units of lattice spacing) is taken to be

(14) E(L)=JL

where $J=a\cdot \sigma_{QCD}$ is the string tension **times the lattice spacing** a, so that J has dimensions of energy. This is the dimensional analog of the QCD string tension $\sigma_{QCD}\approx (440\;MeV{)}^{2}$; the lattice spacing a sets the physical scale. Thus E(L) has dimensions of energy, as required for a thermodynamic free energy.

This flux tube has a finite mass gap Δ∼J, governing the energy cost to rearrange fusion channels via virtual anyon processes [7,8,15].

At finite temperature T, the system is in a mixed state. To describe the thermodynamic behavior of the flux tube, we introduce the following **assumption**: the internal degrees of freedom of the flux tube—described by the $d_{1/2}^{L}$ fusion channels—thermalize at finite temperature, contributing a thermal entropy:

(15) $$S_{th}(L)=\sigma_{k}L+constant$$

where $\sigma_{k}=\ln d_{1/2}$. The justification is that at finite temperature, thermal fluctuations can activate transitions between different fusion channels via intermediate virtual excitations across the mass gap Δ∼J, thereby effectively randomizing the internal color configuration of the flux tube. The number of fusion channels thus provides the density of states for the thermalized transverse excitation modes. Finite-temperature properties of extended string-net models have been investigated in [21].

This assumption applies for T≳Δ, where thermal fluctuations overcome the gap and activate transitions between fusion configurations [12,13,16]. A full dynamical derivation of the thermalization rate from a microscopic Hamiltonian lies beyond the scope of this work. Nevertheless, for T≳Δ, the general principles of statistical mechanics suggest that ergodic dynamics will drive the system toward thermal equilibrium, irrespective of the precise transition rates. The equilibrium distribution is then given by the Boltzmann factor, which depends only on the density of states (the number of fusion channels) and the energy spectrum. Hence, the present assumption suffices for the thermodynamic analysis of the confinement–deconfinement transition. The validity of this identification is not assumed a priori; a proposed protocol for testing it across different categorical structures, system sizes, and boundary conditions is outlined in Appendix A.4.

Therefore, the free energy of the system is

(16) $$F(L,T)=E(L)-TS_{th}(L)=(J-T\sigma_{k})L$$

We note that a constant term independent of L has been omitted as it does not affect the phase transition.

### 4.2. Confinement–Deconfinement Phase Transition

The critical temperature is therefore

(17) $$T_{c}=\frac{J}{\sigma_{k}}=\frac{J}{\ln\left(2\cos\frac{\pi }{k+2}\right)}$$

For the special case k=3 (Fibonacci category), this becomes

(18) $$T_{c}=\frac{J}{\ln\phi }\approx \frac{J}{0.4812}$$

**Nature of the transition**: Because the free energy $F(L,T)=(J-T\sigma_{k})L$ is linear in L, the transition occurs when the coefficient of L changes sign. This corresponds to a **first-order-like** transition (a level crossing or tension sign reversal), rather than a continuous phase transition with critical exponents and scaling laws. The system jumps from the confined phase (L=0) to the deconfined phase (L→∞) at $T_{c}$, with no intermediate phase. This is consistent with the fact that the model has no spatial coupling between different L-segments that could produce critical fluctuations. Accordingly, the term “critical scaling” used in some earlier heuristic discussions is not applicable here; the transition is driven purely by the sign change of the effective tension.

### 4.3. Testability of the Thermalization Assumption

The thermalization assumption introduced above is not an ad hoc postulate; it can be directly tested in numerical simulations of the string-net model. Specifically:

(1) One can compute the microcanonical entropy from the number of fusion channels: $S_{micro}(L)=\ln N(L)$.
(2) One can perform finite-temperature Monte Carlo simulations to extract the thermal entropy $S_{th}(L,T)$.
(3) In the high-temperature limit, one should find $S_{th}(L,T)\to S_{micro}(L)$.

A concrete protocol for testing this in future numerical simulations is provided in Appendix A.

The justification for the thermalization assumption above is that the fusion channels provide the density of states for the transverse excitation modes of the flux tube, and these modes do thermalize at finite temperature. This can be directly tested in numerical simulations of the string-net model (see Appendix A). If further numerical simulations confirm the behavior $S_{th}(L,T)\to S_{micro}(L)$ in the high-temperature limit, then the free energy expression $F(L,T)=(J-T\sigma_{k})L$ becomes a derived result rather than an assumption. The present work focuses on analytic calculations.

### 4.4. Physical Picture of the Phase Transition

This phase transition arises purely from the competition between the energy term and the entropy term in the free energy:

(1) The energy term E=J⋅L tends to shrink the flux tube.
(2) The entropy term $TS=TL\sigma_{k}$ tends to elongate the flux tube (due to more microscopic states).

As the temperature increases, the entropy term gradually dominates and eventually exceeds the energy term at $T_{c}$, leading to the breakdown of confinement. This is a model realization of the entropy surface mechanism proposed by Mamo [3].

## 5. Heuristic Analogy Between $SU(2{)}_{k}$ Fusion Categories and the SU(3) Representation Category

This section provides a structural analogy between $SU(2{)}_{k}$ fusion categories and the representation category of SU(3), in order to illuminate the categorical origin of confinement. It should be understood as a heuristic analogy, not a strict mathematical reduction.

The experimental observation of emergent graviton modes in fractional quantum Hall systems [9] serves as a crucial precedent for the program proposed here. It demonstrates that the emergence of geometric excitations—here, chiral spin-2 modes arising from parton oscillations—is not merely a theoretical construct but an experimentally accessible phenomenon in quantum many-body systems. This lends concrete support to the feasibility of the quantum simulation protocols we outline below.

### 5.1. Comparison of Quantum Dimensions and Fusion Rules

In the SU(3) representation category, the fundamental representation 3 has quantum dimension $d_{3}=3$, the adjoint 8 has dimension 8, etc. In the $SU(2{)}_{k}$ category, the j=1/2 object has quantum dimension $d_{1/2}=2\cos(\pi /(k+2))$, which for k=3 evaluates to ϕ≈1.618. Although the numerical values differ, the algebraic structure of the fusion rules is qualitatively similar.

Table 2 summarizes the key correspondences.

### 5.2. Condition for a Three-Body Singlet

In SU(3), three quarks can form a color singlet (baryon). In the present $SU(2{)}_{k}$ model, however, the fundamental constituent of the flux tube is the half-integer object j=1/2. Due to the $Z_{2}$ grading of $SU(2{)}_{k}$, **any odd number** of j=1/2 objects carries half-integer total charge and **cannot** fuse to the identity **1**, regardless of the value of k. Therefore, a direct analog of the baryonic three-body singlet does not exist for the j=1/2 object in this model. What the model does capture is the **mesonic** channel: a pair of j=1/2 objects fuses to **1**, and by extension, an even-length chain of such objects forms a singlet flux tube. This is sufficient to model the qualitative features of entropic confinement in a $q\bar{q}$ system. A full categorical analog of SU(3) baryons would require a different fusion category (e.g., one containing objects that mimic the (3⊗3⊗3→1 channel), which is left for future work.

### 5.3. Why Use $SU(2{)}_{k}$ Instead of SU(3) Directly?

Although the SU(3) representation category is algebraically closer to QCD, the $SU(2{)}_{k}$ family offers several advantages:

- **Exact solvability**: closed-form expressions for quantum dimensions, fusion-channel counts, and entropy density.
- **Tunable parameter**: k takes integer values from 2 to ∞, allowing study of how confinement properties evolve with the degree of non-Abelianness.
- **Qualitative consistency with lattice estimates**: both k=2 and k=3 entropy densities lie within the reference range which are of the same order of magnitude as the estimated entropy density from lattice simulations.
- **Fibonacci case**: k=3 reduces to the Fibonacci category, often viewed as the minimal non-Abelian truncation of the SU(3) representation category.

Extending the present model to the genuine SU(3) representation category—distinguishing 3 from $\bar{3}$ and incorporating higher-dimensional representations such as the adjoint 8—is left for future work.

We emphasize that this is a heuristic structural analogy, not a rigorous mathematical equivalence between the two categories. The simplification is necessary to achieve analytic solvability for flux-tube entanglement entropy.

### 5.4. Further Numerical Comparisons and the Minimal Truncation

The quantum dimensions in the SU(3) representation category are $d_{3}=3$, $d_{8}=8$, $d_{10}=10$, etc., which are significantly larger than the quantum dimensions in $SU(2{)}_{k}$ (e.g., $d_{1/2}=2\cos\left(\frac{\pi }{k+2}\right)$). Despite these numerical differences, the fusion rule structures share key qualitative features.

As noted in Section 5.2, the $Z_{2}$ grading of $SU(2{)}_{k}$ forbids odd-length chains of j=1/2 objects from fusing to the identity. Consequently, **a direct analog of the SU(3) baryonic three-body singlet does not exist in this model.** What the model does capture is the mesonic channel: pairs of j=1/2 objects fuse to **1**, and by extension, even-length chains form singlet flux tubes. The Fibonacci category k=3 remains the simplest non-Abelian fusion category that realizes this mesonic confinement structure. This justifies viewing $SU(2{)}_{3}$ as a minimal non-Abelian truncation of the SU(3) representation category for the mesonic (pairing) sector, after neglecting differences in representation dimensions and the distinction between 3 and $\bar{3}$.

## 6. Discussion

### 6.1. The Parameter k as a Theoretical Control Knob

A key advantage of the $SU(2{)}_{k}$ framework is that the parameter k provides a continuous tuning knob for the entropy density $\sigma_{k}=\ln\left(2\cos\frac{\pi }{k+2}\right)$. As k increases from 2 to ∞, $\sigma_{k}$ increases monotonically from $\ln\sqrt{2}\approx 0.347$ to ln2≈0.693. This allows systematic study of how the entropic force driving deconfinement, characterized by $T_{c}=J/\sigma_{k}$, depends on the categorical structure. For k=3, the model reduces to the Fibonacci category, which provides the minimal non-Abelian realization.

### 6.2. Relation to the Fibonacci Category

When k=3, the $SU(2{)}_{3}$ fusion category reduces to the Fibonacci category. In this limit, our results reproduce those of the Fibonacci model: the entropy density σ=lnϕ≈0.48 and the critical temperature $T_{c}=J/\ln\phi$. The Fibonacci category is the simplest rank-2 non-Abelian fusion category, and its appearance in the k=3 case suggests that the essential features of entropic confinement—linear entropy growth and a confinement–deconfinement phase transition—do not require the full structure of $SU(2{)}_{k}$ but only a non-Abelian fusion rule where a nontrivial object fuses with itself to the identity.

### 6.3. Physical Significance of the Entropy Density lnϕ

The golden ratio $\phi =(1+\sqrt{5})/2$ is the hallmark constant of the Fibonacci category, originating from the fusion rule τ⊗τ=1⊕τ. The Fibonacci category is also the basis for non-Abelian anyon models [13]. Even though current lattice estimates cannot distinguish between $\sigma =\ln\sqrt{2}\approx 0.347$ and lnϕ≈0.48, it is instructive to explore the hypothetical scenario where $\sigma_{QCD}$ were exactly lnϕ. In that case, it might imply:

(1) **Universal non-Abelian structure:** The SU(3) representation category and the Fibonacci category share a qualitative feature in their fusion rules: the fusion of two nontrivial objects contains the singlet, and the fusion of three nontrivial objects also contains the singlet. This structure implies that a nontrivial object (3 in SU(3), τ in Fibonacci) cannot exist in isolation but must pair to form a singlet—precisely the categorical manifestation of confinement. The Fibonacci category may be viewed as the minimal non-Abelian truncation of the SU(3) representation category after neglecting differences in representation dimensions.
(2) **Universality of color entanglement entropy**: The flux-tube entanglement entropy densities of different gauge groups (SU(2), SU(3), SU(N)) might be described by some universal function, and the Fibonacci category corresponds to the value of this universal function in the minimal non-Abelian realization.

However, this remains speculative pending more precise lattice calculations.

### 6.4. Model Support for the Entropy Surface Mechanism

The entropy surface mechanism proposed by Mamo [3] argues that confinement arises from the sign reversal of the entanglement entropy gradient at a critical radius $R_{EE}$. Our model provides a concrete realization for this mechanism:

In the free energy $F=(J-T\sigma_{k})L$, the entropy term TS grows linearly with L, generating a driving force opposite to the energy term.

(1) When $T<T_{c}$, the free energy increases with L, equivalent to a negative entropy gradient (confinement).
(2) When $T>T_{c}$, the free energy decreases with L, equivalent to a positive entropy gradient (deconfinement).

Therefore, our model provides a concrete analog realization of this mechanism within $SU(2{)}_{k}$ fusion categories, providing a computational framework for understanding the microscopic origin of the entropy gradient sign reversal.

### 6.5. Comparison with Related Models

This section compares the $SU(2{)}_{k}$ fusion category model with other relevant theoretical models to clarify similarities, differences, and advantages.

(1) **Ising category (**k=2 **special case)**

For k=2, the $SU(2{)}_{2}$ fusion category reduces to the Ising category. Its nontrivial object σ (corresponding to j=1/2) has quantum dimension $d_{\sigma }=\sqrt{2}$, giving an entropy density $\sigma_{2}=\ln\sqrt{2}\approx 0.347$. The fusion rule σ⊗σ=1⊕ψ (where ψ is a Majorana fermion) does not contain the identity when fusing three σ anyons; therefore the Ising category cannot reproduce a baryon-like (three-quark) singlet. The Ising category thus does not support a baryon-like singlet. This is consistent with the $Z_{2}$ grading of $SU(2{)}_{k}$ discussed in Section 2.3 and Section 5.2. Nevertheless, the Ising category serves as a valuable testbed for computational methods in string-net models as the simplest non-Abelian fusion category.

(2) **Fibonacci category (**k=3 **special case)**

For k=3, the $SU(2{)}_{3}$ category reduces to the Fibonacci category, which is the most interesting case for this work. The nontrivial object τ has quantum dimension equal to the golden ratio ϕ≈1.618, leading to an entropy density $\sigma_{3}=\ln\phi \approx 0.481$. The fusion rule τ⊗τ=1⊕τ is the defining relation of the Fibonacci category. In the j=1 subcategory of $SU(2{)}_{3}$, three τ’s can indeed fuse to the identity; however, this does not constitute a baryon-like singlet in our j=1/2 flux-tube model, where the $Z_{2}$ grading forbids odd-length chains. The Fibonacci category is the simplest rank-2 non-Abelian fusion category, and its entropy density lies in the same order of magnitude as the QCD entropy density estimated from the entropy surface mechanism and is of the same order of magnitude as lattice estimates. Although this numerical agreement may be coincidental, the Fibonacci category is often viewed as the minimal non-Abelian truncation of the SU(3) representation category.

(3) **General **$SU(2{)}_{k}$ **categories (**k>3**)**

For k>3, the category contains more simple objects (j=0,1/2,1,3/2,…,k/2). The entropy density $\sigma_{k}=\ln(2\cos\frac{\pi }{k+2})$ increases monotonically with k, from $\ln\sqrt{2}\approx 0.347$ at k=2 to ln2≈0.693 as k→∞. The parameter k thus provides a continuous tuning knob for the entropy density, allowing systematic study of how the confinement–deconfinement phase transition depends on the algebraic structure. For instance, the critical temperature $T_{c}=J/\sigma_{k}$ decreases with increasing k, implying that richer internal degrees of freedom favor deconfinement. This parametric dependence can be tested in artificial topological systems such as quantum simulators.

(4) **Other string-net models and topological entanglement entropy**

The Levin–Wen string–net model [7,8] provides a general framework for constructing ground states of topological orders. The ground-state topological entanglement entropy was originally defined in [7,8], and the string-net condensation mechanism was developed in [15]. The Rényi entropies of string-net models have also been studied in [22]. However, the topological entanglement entropy computed for the ground state is a constant (e.g., the Kitaev-Preskill or Levin–Wen topological entropy), not a quantity that grows with system size. The present work focuses on the entanglement entropy of an excited state (the flux tube), rather than the ground state. This distinction is crucial: the ground-state topological entropy is constant and independent of length, while the entanglement entropy of the internal degrees of freedom of the flux tube grows linearly with length, which is precisely the manifestation of the entropic nature of confinement.

(5) **Entropic confinement models in QCD**

The entropy surface mechanism proposed by Mamo [3] argues that confinement originates from a sign reversal of the entanglement entropy gradient at a critical radius $R_{EE}$. Our model provides a concrete realization of this mechanism through the free energy competition $F=(J-T\sigma_{k})$: for $T<T_{c}$ the free energy increases with L (negative entropy gradient, confinement), while for $T>T_{c}$, it decreases with L (positive entropy gradient, deconfinement). Unlike the phenomenological analysis of Mamo, our model computes the entropy density $\sigma_{k}$ exactly from first principles of fusion categories and directly links the sign reversal of the entropy gradient to the critical temperature $T_{c}=J/\sigma_{k}$. Thus, it offers an exactly solvable analogy-model microscopic realization of the entropy surface mechanism.

(6) **Advantages and limitations of the present model**

**Advantages:** (1) Exact solvability—$\sigma_{k}$ is given by a closed-form expression; (2) tunable parameter—k continuously controls the non-Abelian nature; (3) qualitative agreement with lattice estimates—both k=2 and k=3 entropy densities lie within the reference range which is of the same order of magnitude as the estimated entropy density from lattice simulations; and (4) testable predictions—the entropy jump, inverse mass gap–correlation length relation, etc., can be tested in quantum simulators.

**Limitations:** (1) Degenerating SU(3)’s 3 and $\bar{3}$ into the same object j=1/2; (2) ignoring higher-dimensional representations such as the adjoint 8; and (3) omitting dynamical fermions and chiral symmetry breaking. Overcoming these limitations requires generalization to the SU(3) representation category.

### 6.6. Testable Predictions

Although highly simplified, our model can still propose several qualitative predictions that can be tested in lattice, string-net model simulations, or heavy-ion collision experiments:

(1) **Entropy density dependence on **k: For different k (corresponding to different topological orders), the flux-tube entanglement entropy density $\sigma_{k}=\ln\left(2\cos\frac{\pi }{k+2}\right)$ increases monotonically with k. This dependence can be tested in artificial topological systems such as quantum simulators.
(2) **Entropy jump at the transition temperature**: At the deconfinement phase transition temperature $T_{c}=J/\sigma_{k}$, the flux-tube entanglement entropy should exhibit a sudden change in its slope $dS_{flux}/dL$ of magnitude $\sigma_{k}$ (jumping from 0 in the confined phase to $\sigma_{k}$ in the deconfined phase).
(3) **Universal behavior for different gauge groups**: For SU(N) gauge theories, the flux-tube entanglement entropy density $\sigma_{N}$ should approach some asymptotic value as N increases. This could serve as a reference value for the low-energy effective description of SU(3).

This work treats the SU(2)_k_ model as a minimal solvable toy model for exploring entropic confinement, rather than a direct realization of full QCD.

### 6.7. Open Question: Reference State of Entropy

Throughout this paper, we have implicitly taken the vacuum (**1**) of the $SU(2{)}_{k}$ category as the fixed reference state for entropy calculations. While this choice is natural for a topologically ordered system, it prompts a deeper question: In a dynamical theory like QCD, what is the correct reference state with respect to which entropy is defined? Furthermore, can this reference state itself evolve, undergo phase transitions, or even become a quantum superfluid?

In our model, the confinement–deconfinement transition is driven by the competition $F=(J-T\sigma_{k})L$, which implicitly assumes a fixed entropy baseline. However, in the full QCD vacuum, the reference state of the confined phase (the non-perturbative QCD vacuum) and that of the deconfined phase (the Quark-Gluon Plasma, or QGP) have vastly different entropy densities. The QGP is not just a high-temperature excitation above the same vacuum; it is a qualitatively new reference state.

This line of reasoning suggests a radical possibility: confinement and deconfinement might represent different “reference states of entropy” rather than merely different excitation spectra above a single, fixed vacuum. The phase transition between them would then correspond to a sudden change in the entropy baseline itself. This perspective resonates with the holographic principle, where entropy is primarily a property of the horizon, and with topological order, where different phases have distinct reference states characterized by different total quantum dimensions. We plan to explore this direction in future work.

### 6.8. Limitations and Future Work

The main limitations of this model include:

(1) Degenerating SU(3)’s 3 and $\bar{3}$ into the same object j=1/2, ignoring the distinction between color charge and anticharge.
(2) Ignoring contributions from higher-dimensional representations such as the adjoint 8.
(3) Not considering important QCD features such as quark mass and chiral symmetry breaking.

Overcoming these limitations requires generalizing to richer fusion categories. Generalizations to other fusion categories are discussed in [23,24].

Future work can proceed in the following directions:

(1) **Generalization to SU(3) representation categories**: Extend the model to the real fusion rules of SU(3) (distinguishing 3 and $\bar{3}$) and explore whether more precise QCD phenomenology can be obtained.
(2) **Lattice verification:** Perform numerical simulations in the $SU(2{)}_{k}$ string-net model to compute the relationship between entropy correlation length and mass gap, verifying Δ∼1/ξs.
(3) **Non-equilibrium categorical dynamics**: A systematic non-equilibrium statistical mechanics framework for fusion categories has been developed in a separate work [25], where master equations on the $SU(2{)}_{k}$ fusion category are derived and the entropy production rate is proven to be non-negative. Entanglement renormalization for string-net models was studied in [26]. Extending the present equilibrium analysis to include such non-equilibrium dynamics would provide a more complete description of the thermalization of flux-tube fusion channels and the entropy production during the confinement–deconfinement transition.
(4) **Generalization to richer categorical structures**: Explore extending similar methods to categorical structures closer to QCD, such as appropriate truncations of the SU(3) representation category. The methodological framework developed here can be generalized to richer fusion categories [15]. The anyon-chain construction based on fusion rules, as discussed in Ref. [27], provides a natural starting point for such generalizations.

## 7. Conclusions and Outlook

### 7.1. Conclusions

In this paper, we have proposed a heuristic demonstration model based on $SU(2{)}_{k}$ fusion categories to understand the entropic nature of QCD color confinement. The main conclusions are as follows:

(1) Confinement is a direct consequence of fusion rules: In $SU(2{)}_{k}$ fusion categories, the nontrivial object j=1/2 cannot exist in isolation but must pair to form singlets. This is completely analogous to color confinement in the SU(3) representation category.
(2) The flux-tube entanglement entropy density is $\sigma_{k}=\ln\left(2\cos\frac{\pi }{k+2}\right)$: The number of fusion channels grows exponentially with length, and the entropy density is given by the logarithm of the quantum dimension $d_{1/2}.For\;k=3,$ this reduces to lnϕ≈0.4812, which is of the same order of magnitude as the entropy density scale inferred from lattice simulations and the entropy surface mechanism [3].
(3) Competition between energy and entropy drives the deconfinement phase transition: Under the assumption that fusion channels thermalize at finite temperature, the free energy $F=(J-T\sigma_{k})L$ changes sign at $T_{c}=J/\sigma_{k}$, marking the transition from the confined phase to the deconfined phase. This is a first-order-like transition (tension sign reversal), consistent with the linear dependence of the free energy on L.
(4) The parameter k provides a theoretical control knob: The entropy density $\sigma_{k}$ takes integer values with k, allowing systematic study of entropic confinement across different categorical structures.

This model provides a minimal realization of the entropic confinement mechanism and suggests that the color degrees of freedom in QCD might be a low-energy manifestation of some fusion category. The heuristic consistency of lnϕ≈0.48 with the estimated entropy density from lattice simulations and the entropy surface mechanism, while likely coincidental, hint at a deeper connection between non-Abelian fusion categories and entropic confinement mechanisms.

Our categorical approach may also provide insights into the QCD phase diagram relevant for heavy-ion collision experiments, where the entropy density of the flux tube could be related to the excess entropy production in the deconfinement transition. Moreover, the tunable parameter k in the $SU(2{)}_{k}$ model suggests that analogous fusion categories could be realized in cold-atom quantum simulators, offering a controllable laboratory for studying non-Abelian confinement phenomena in nuclear physics.

### 7.2. Outlook

The framework developed in this work—the competition between quantum entropy and enthalpy—is rooted in the fundamental thermodynamic relation F=U−TS. If we view the universe as a quantum system at its deepest level, this competition becomes a universal principle governing the evolution, structure formation, and phase transitions of quantum systems. Preliminary results along these directions have been reported in Refs. [25,28,29].

To describe multi-level interactions between a quantum system and its subsystems, higher category theory offers a natural language that can handle hierarchical structures and relational mappings. Several directions are being pursued based on this framework:

(1) **SU(3) representation categories**: extending the model to genuine SU(3) fusion rules to capture both 3 and $\bar{3}$ sectors;
(2) **Non-equilibrium dynamics**: incorporating master equations on fusion categories to study entropy production during the confinement–deconfinement transition;
(3) **Cosmological applications**: exploring the entropy–enthalpy competition mechanism in the QCD phase transition of the early universe and in dark-energy modeling.

More systematic theoretical development and numerical verification will constitute the core of our future research.

## Figures and Tables

**Table 1:** Entropy density $\sigma_{k}$ for selected k values.

k	$d_{1/2}$	$\sigma_{k}=\ln d_{1/2}$
2	$2\cos(\pi /4)=\sqrt{2}\approx 1.414$	≈0.3466
3	2cos(π/5)=ϕ≈1.618	≈0.481
4	$2\cos(\pi /6)=\sqrt{3}\approx 1.732$	≈0.549
k→∞, (limit)	2cos(0)=2	ln2≈0.6931

**Table 2:** Analogy between $SU(2{)}_{k}$ fusion categories and the SU(3) representation category.

SU(3) Representation Category	$SU(2{)}_{k}$ Fusion Category
Fundamental 3 (color charge)	Object j=1/2
Antifundamental $\bar{3}$	Same object j=1/2 (not distinguished in this minimal model)
Meson singlet: $3⊗\bar{3}=1⊕8$	Meson singlet: $\frac{1}{2}⊗\frac{1}{2}=0⊕1$
Baryon singlet: 3⊗3⊗3=1⊕⋯	Flux tube: even-length chain of 1/2’s fusing to 1
Confinement: 3 cannot appear in isolation	Confinement: j=1/2 cannot appear in isolation

## Data Availability

This is an analytical study; no simulation data were generated. A protocol for future numerical verification is given in Appendix A.

## Appendix A. Proposed Numerical Protocol for the $SU(2{)}_{k}$ String-Net Model

The string-net model and flux-tube construction follow the definitions in Section 2.5. This appendix outlines a numerical verification scheme for the flux-tube entanglement entropy in the $SU(2{)}_{k}$ string-net model, demonstrating the verifiability of the analytical results presented in the main text.

### Appendix A.1. Model and Algorithm

Define the ground-state wave function of the Levin–Wen string-net model on a two-dimensional lattice (e.g., a triangular lattice) [7,8], where the string labels are taken from the $SU(2{)}_{k}$ fusion category (j=0,1/2,1,…,k/2). The ground-state wave function can be constructed explicitly via string-net condensation [13,14], or alternatively simulated using tensor network methods such as PEPS (projected entangled pair states). For PEPS-based simulations, one would set the bond dimension to D = 8–16 to control truncation error in the entanglement entropy calculations described below.

To introduce a flux tube, create a pair of j=1/2 anyons as defects at lattice sites i and j, separated by distance L=|i−j|. Within the topological sector that fixes these two anyons, the minimum energy state of the system corresponds to the flux tube connecting the two defects. Its wave function can be obtained by imposing fixed topological boundary conditions on the string-net.

In practice, we use a **triangular lattice with open boundaries**, lattice spacing a=1, and system sizes L=4,6,8…20 to avoid finite-size artifacts. The ground-state is prepared via string-net condensation, and flux-tube defects are inserted at fixed separation L.

### Appendix A.2. Entanglement Entropy Calculation

Consider a region that contains a segment of the flux tube of length L and compute its entanglement entropy S(L). According to Section 3.3

S(L)=lnN(L)

where N(L) is the number of fusion channels. This is an exact relation. The asymptotic behavior for L≫1 is given below.

$$S(L)\approx L\ln d_{1/2}+constant$$

from which the entropy density $\sigma_{k}=\ln d_{1/2}$ can be extracted. For the Fibonacci case (k=3), the constant term can be fixed exactly. Since $N(L)=F_{L-1}$ (the Fibonacci numbers) with asymptotic behavior $F_{L-1}∼\phi^{L-1}/\sqrt{5}$, we have

$$S(L)=\ln N(L)\approx L\ln\phi -\ln(\phi \sqrt{5})$$

up to subleading corrections. For general k, the constant term depends on the subleading eigenvalue of the transfer matrix; its exact form is not needed for extracting the entropy density $\sigma_{k}=\ln d_{1/2}$, which is determined solely by the largest eigenvalue $d_{1/2}$.

The entanglement entropy S(L) is computed for a **slab region enclosing the central** L/2 **sites** of the flux tube, with PEPS bond dimension D = 8–16 to control truncation error below $10^{-3}$.

### Appendix A.3. Finite-Temperature Simulation

In principle, finite temperature T can be introduced via imaginary-time path integrals, and the partition function $Z(L,T)=Tr(e^{-\beta H})$ can be computed, yielding the free energy F(L,T)=−TlnZ(L,T). Numerical simulations should verify whether the free energy satisfies the predicted form:

$$F(L,T)=(J-T\sigma_{k})L$$

and thereby determine the critical temperature $T_{c}=J/\sigma_{k}$. Near $T_{c}$, the rate of change of the entanglement entropy S(L) with respect to L should exhibit a jump of magnitude $\sigma_{k}$.

One would perform imaginary-time evolution with step size δβ=0.05 and $N_{\tau }$ = 200–500 steps. The free energy is extracted via $F=-\beta^{-1}\ln Z$, and $T_{c}$ is determined from the crossing point of F/L curves at different L. To reliably identify the crossing point, one would compute F(L,T)/L for at least four system sizes, e.g., L=4,6,8,10. For each temperature T, we plot F(L,T)/L as a function of $1/L^{2}$ and extrapolate to the thermodynamic limit (L→∞) using a linear fit. The critical temperature $T_{c}$ is then identified as the temperature at which the extrapolated values for different L intersect (or, equivalently, the temperature at which the F/L curves for different L cross). This finite-size scaling procedure follows the standard approach for determining phase transition temperatures in lattice systems. To estimate the uncertainty in Tc, one would repeat the procedure for multiple independent Monte Carlo runs and take the standard deviation of the resulting Tc values.

### Appendix A.4. Proposed Test of the Thermalization Assumption

A crucial test of our thermalization assumption (Section 4.1) is to compare:

(1) The microcanonical entropy computed from fusion channels: $S_{micro}(L)=\ln N(L)$.
(2) The thermal entropy extracted from finite-temperature simulations: $S_{th}(L,T)$.

If, in the high-temperature limit $S_{th}(L,T)\to S_{micro}(L)$, the thermalization assumption is justified.

**Method**: For each parameter set (k,L), we compute $S_{micro}(k,L)$ exactly from the fusion rules of the $SU(2{)}_{k}$ category. The thermal entropy $S_{th}(k,L,T)$ is extracted from finite-temperature path-integral simulations of the string-net model. The flux-tube mass gap is $\Delta_{k}=J$ for all k≥2, dominated by the string tension (see Section 4.1).

**Criterion**: Thermalization is considered verified if, for $T\geq 2\Delta_{k}$,

$$\frac{\left|S_{th}-S_{micro}\right|}{S_{micro}}<5\%$$

If the assumption holds, one would expect that for $T\geq 2\Delta_{k}$, the thermal entropy $S_{th}(L,T)$ converges to the microcanonical entropy $S_{micro}(L)$ with deviations below 5%. This convergence would confirm that the identification $S_{th}≃S_{micro}$ is robust across different categorical structures, system sizes, and boundary conditions, thereby justifying its use in the free energy minimization of Section 4.

### Appendix A.5. Comparison with Lattice Results

If performed, the simulation results could be compared with lattice QCD measurements of FTE^2^ [4,5]:

(1) The entropy densities $\sigma_{2}=\ln\sqrt{2}\approx 0.347$ and $\sigma_{3}=\ln\phi \approx 0.4812$ are of the same order of magnitude as the reference scale inferred from lattice data [4,5] and the entropy surface mechanism [3] (see the Introduction for details).
(2) The entropy correlation length $\xi_{s}$ (defined via entropy-entropy correlation functions) and the mass gap Δ (the minimum energy to excite a j=1/2 pair) should satisfy $\Delta \xi_{s}∼1$.
(3) The critical behavior of the deconfinement phase transition shows qualitatively consistent with that of SU(3) pure gauge theory.

The entropy correlation length $\xi_{s}$ is extracted by fitting $S(r)∼e^{-r/\xi_{s}}$, and the mass gap (Δ) is obtained from the lowest excitation energy of j=1/2 pairs. We check the scaling $\Delta \xi_{s}$ within 10% error.

### Appendix A.6. Generalization to Richer Categorical Structures

As a future direction, one may explore extending similar methods to categorical structures closer to QCD, such as appropriate truncations of the SU(3) representation category, and directly compute the entanglement entropy of the corresponding flux tubes for comparison with the $SU(2{)}_{k}$ model results.

## References

[1] Greensite J. (2003). The confinement problem in lattice gauge theory. Prog. Part. Nucl. Phys..

[2] Ogilvie M.C. (2011). Quark confinement and the renormalization group. Philos. Trans..

[3] Mamo K.A. (2025). Entanglement, trace anomaly, and confinement in QCD. Phys. Rev. D..

[4] Amorosso R., Syritsyn S., Venugopalan R. (2024). Entanglement entropy of a color flux tube in (2+1)D Yang-Mills theory. J. High Energy Phys..

[5] Amorosso R., Syritsyn S., Venugopalan R. (2025). Internal color contributions to flux tube entanglement entropy. Proc. Sci..

[6] Takahashi T.T., Kanada-En’yo Y. (2019). Lattice QCD study of static quark and antiquark correlations via entanglement entropies. Phys. Rev. D..

[7] Kitaev A., Preskill J. (2006). Topological entanglement entropy. Phys. Rev. Lett..

[8] Levin M., Wen X.-G. (2006). Detecting topological order in a ground state wave function. Phys. Rev. Lett..

[9] Yang Z., Wang Y., Lu X., Liu Z., Baldwin K.W., West K.W., Pfeiffer L.N., Yang B., Balram A.C., Du L. (2026). Emergent partons in fractional quantum Hall systems. Nat. Phys..

[10] Yang X.D., Yang Y.C., Mei H.L. (2026). Quark Confinement as an Entropic Phenomenon: Insights from the Fibonacci Category. Rep. Adv. Phys. Sci..

[11] Di Francesco P., Saleur H., Zuber J.-B. (1987). Modular invariance in non-minimal two-dimensional conformal theories. Nucl. Phys. B.

[12] Slingerland J.K., Bais F.A. (2001). Quantum groups and non-Abelian braiding in quantum Hall systems. Nucl. Phys. B.

[13] Preskill J. (2004). Chapter 9: Topological quantum computation. Lecture Notes for Physics 219: Quantum Computation.

[14] Bonesteel N.E., Hormozi L., Zikos G., Simon S.H. (2005). Braid Topologies for Quantum Computation. Phys. Rev. Lett..

[15] Levin M.A., Wen X.-G. (2005). String-net condensation: A physical mechanism for topological phases. Phys. Rev. B.

[16] Wen X.-G. (2007). Quantum Field Theory of Many-Body Systems: From the Origin of Sound to an Origin of Light and Electrons. Oxford Graduate Texts.

[17] Green D., Huston P., Kawagoe K., Penneys D., Poudel A., Sanford S. (2024). Enriched string-net models and their excitations. Quantum.

[18] Kong L. (2014). Anyon condensation and tensor categories. Nucl. Phys. B.

[19] Rabenstein A., Bodendorfer N., Schäfer A., Buividovich P. (2019). Lattice study of Rényi entanglement entropy in lattice Yang-Mills theory. Phys. Rev. D..

[20] Raychowdhury I., Stryker J.R. (2020). Loop, string, and hadron dynamics in SU(2) Hamiltonian lattice gauge theories. Phys. Rev. D..

[21] Soares A., Ritz-Zwilling A., Fuchs J.-N. (2025). Extended string-net models with all anyons at finite temperature. Phys. Rev. B.

[22] Hermanns M., Trebst S. (2014). Rényi entropies for classical string-net models. Phys. Rev. B.

[23] Lin C.-H., Burnell F.J. (2024). Anyon condensation in string-net models. Phys. Rev. B.

[24] Hahn A., Wolf R. (2020). Generalized string-nets for unitary fusion categories without tetrahedral symmetry. Phys. Rev. B.

[25] Yang X.D., Yang Y.C., Mei H.L. (2026). Non-equilibrium categorical dynamics: Entropy, enthalpy, and the approach to confinement. Phys. Lett. A.

[26] König R., Reichardt B.W., Vidal G. (2009). Exact entanglement renormalization for string-net models. Phys. Rev. B.

[27] Finch P.E., Flohr M., Frahm H. (2014). Integrable anyon chains: From fusion rules to face models to effective field theories. Nucl. Phys. B.

[28] Yang X.D., Yang Y.C., Mei H.L. (2026). Antimatter generation mechanism: A new perspective from entropy flow vector based on the quantum tensor network theory. Front. Phys..

[29] Yang X.D., Yang Y.C., Mei H.L. (2026). Spacetime as emergent order: A testable framework from string-net condensation to geometric thermodynamics. Front. Astron. Space Sci..

